# Editorial: Pulmonary embolism and pulmonary hypertension: from acute management to long-term phenotyping

**DOI:** 10.3389/fcvm.2026.1851852

**Published:** 2026-05-08

**Authors:** Hugo Hyung Bok Yoo, Andi Rroku, Tharusan Thevathasan

**Affiliations:** 1 Pulmonary Division, São Paulo State University (UNESP)-Botucatu Medical School, Botucatu, São Paulo, Brazil; 2 Deutsches Zentrum für Herz-Kreislauf-Forschung (DZHK), Berlin, Germany; 3 Department of Cardiology, Angiology and Intensive Care Medicine, Deutsches Herzzentrum der Charité (DHZC), Campus Benjamin Franklin, Berlin, Germany; 4 Berlin Institute of Health (BIH), Berlin, Germany

**Keywords:** clinical phenotyping, long-term outcomes, pulmonary embolism, pulmonary hypertension, pulmonary vascular diseases

Pulmonary embolism (PE) and pulmonary hypertension (PH) remain important causes of complications and life-threatening risks in various clinical settings (1). These conditions involve complex pathophysiological mechanisms, present a variety of symptoms, and pose considerable challenges for diagnosis, risk assessment, and management. Even with significant advances in recent years, many questions remain unanswered, especially regarding the best way to select patients for specific interventions, improve long-term outcomes, and better understand the biology of these diseases (2). The studies included in this *Research Topic* illustrate well the current stage of the field. Rather than presenting one major advance, they offer a set of clinically relevant contributions that help to refine current understanding and management of pulmonary vascular diseases. A particularly notable theme is the increasing focus on follow-up, persistent symptoms, and individualized care beyond the acute phase.

A further point of growing and controversial debate in acute PE management concerns the optimal therapeutic approach to patients classified as intermediate to high-risk. Huang and Chen reported similar efficacy and safety between catheter-directed urokinase thrombolysis and rapid intraprocedural alteplase infusion. Beyond the procedural comparison itself, their findings suggest a possible practical advantage, since shorter catheter dwell time may facilitate procedural efficiency in real-world settings. However, these results should be interpreted with caution, as the study is limited by its retrospective single-center design and a small, imbalanced sample size—particularly in the alteplase group—which may introduce selection bias and limit generalizability.

In a retrospective cohort study, the outcomes reported by Senosy et al. were promising. They compared ultrasound-assisted catheter-directed thrombolysis (EKOS) with systemic alteplase and observed similar recovery of right ventricular function and hemodynamic improvement, but with fewer major bleeding events in the catheter-directed group. Although it may not be available at all centers managing acute PE, EKOS may represent a therapeutic option in carefully selected patients with intermediate- to high-risk PE. More robust randomized evidence from the HI-PEITHO trial provides additional context, demonstrating that ultrasound-assisted catheter-directed thrombolysis reduced early hemodynamic decompensation compared with anticoagulation alone, without a clear mortality benefit and with ongoing concerns regarding bleeding risk (3).

The long-term outcomes following acute PE were also addressed in this Research Topic. Wang et al. evaluated right ventricular recovery during follow-up in patients with intermediate-risk PE and reported more favorable hemodynamic status among those treated with reperfusion, particularly catheter-directed thrombectomy, compared with anticoagulation alone. However, given its single-center retrospective design, small sample size, use of domestic devices, and low-dose alteplase for thrombolysis, these findings should be interpreted with caution and require further well-designed studies. Moreover, the absence of standardized follow-up imaging raises the possibility of residual confounding and measurement bias, precluding definitive patient-centered or clinical outcome benefit beyond surrogate hemodynamic improvements.

The collection also appropriately highlighted survivorship after PE. In a large longitudinal cohort, Kirchberger et al. showed that dyspnea following a first PE is common and frequently persistent, with a clear impact on health-related quality of life. Importantly, dyspnea appears to reflect more than residual physiological impairment, as it is also closely associated with depression and anxiety. Nonetheless, the lack of systematic cardiopulmonary functional assessment limits the ability to disentangle the relative contributions of physiological and psychological factors.

Overall, these findings highlight that recovery after acute PE is often incomplete, an aspect still underestimated in clinical practice. For many patients, symptoms persist, functional capacity remains limited, and the impact of disease extends well beyond the acute phase. This has important implications for care. Follow-up strategies that focus primarily on recurrence and anticoagulation are unlikely to fully address patients’ needs. This highlights a critical gap in current care models and supports the need for structured, multidimensional follow-up strategies.

Risk stratification is also still evolving, particularly in predicting risk of developing venous thromboembolism (VTE) in patients with lung cancer. According to a retrospective study by Dougherty et al., the Khorana Score may offer limited discriminatory value in heterogeneous groups of patients with lung cancer, suggesting that risk assessment of VTE could be improved by incorporating more disease-specific features, such as histological subtype and oncogenic driver status. This also serves as an important reminder that prediction models based mainly on general clinical variables may not perform consistently well in different clinical settings. In the future, more refined approaches that better reflect the biological complexity of the disease will likely be necessary. However, the absence of prospective validation and standardized biomarker integration limits the immediate clinical applicability of these approaches. Future strategies will likely require integration of clinical, molecular, and disease-specific features to enable more precise risk stratification.

An additional strength of this Research Topic is the inclusion of pulmonary hypertension associated with chronic obstructive pulmonary disease (PH-COPD). In their review, Scabello et al. addressed a common and clinically important condition that remains underrecognized and lacks evidence-based treatment strategies. By discussing the concept of a pulmonary vascular phenotype in COPD and presenting real-world data from a Brazilian referral center suggesting lower prevalence than previously reported, the authors challenge some established assumptions. Their contribution highlights the need for improved phenotyping and stronger evidence to guide treatment decisions in this population.

The case reports included in this collection provide important clinical insights. Xue et al. description of paradoxical embolism in a patient with thrombophilia underscores the importance of thorough diagnostic reasoning. Specifically, it highlights that clinicians should investigate underlying thrombophilia when VTE persists despite ongoing treatment. Furthermore, the authors emphasize the importance of genetic testing to improve the diagnosis and treatment strategies. However, as a single case report, these findings remain hypothesis-generating, and the clinical utility and cost-effectiveness of routine genetic testing require further validation.

In a parallel contribution, Wang et al. reported a severe case of PH affecting both mother and newborn. Although their clinical features overlapped, the underlying mechanisms were entirely different. In the mother, severe PH was caused by a rare ACVRL1 gene mutation, which was worsened by the physiological stress of pregnancy. In contrast, the newborn's condition represented a reversible response to an adverse intrauterine environment. This case illustrates the importance of multidisciplinary evaluation when PH occurs in the context of pregnancy, genetic disease, and neonatal care. However, the highly specific clinical context and rarity of the underlying condition limit the generalizability of these observations.

Taken together, the studies included in this Research Topic suggest that progress in PE and PH management will depend not only on the development of new therapeutic approaches but also on the adoption of a more integrated and patient-centered perspective across the entire disease trajectory. Bridging acute management with longitudinal follow-up, phenotypic characterization, and biological insight will be essential to meaningfully improve outcomes in this complex population.

We thank the authors for their valuable contributions, the reviewers for their careful work, and the *Frontiers in Cardiovascular Medicine* Editorial Team for their support and for the opportunity to contribute. We hope this collection will stimulate future research in pulmonary vascular diseases where significant uncertainties still persist.
